# Use of artesunate in the treatment of severe imported malaria in France: review of the effectiveness and real-life safety in two French university hospitals

**DOI:** 10.1186/s12879-023-08260-6

**Published:** 2023-05-25

**Authors:** M. Bonsergent, M. Tching-Sin, S. Honoré, P. Bertault-Peres, A. Lepelletier, L. Flet, T. Perez

**Affiliations:** 1 Nantes Université, CHU Nantes, Pharmacie, Nantes, F-44000 France; 2 Assistance Publique des Hôpitaux de Marseille, CHU Marseille, Pharmacie, Marseille, France

**Keywords:** Artesunate, Malaria, Safety, Imported malaria, Adverse events

## Abstract

**Background:**

Intravenous artesunate (AS) is the first-line treatment for patients with severe imported malaria (SIM) worldwide. However, after 10 years of use in France, AS hasn’t yet received marketing authorization.The purpose of this study was to assess the real-life effectiveness and safety of AS in the treatment of SIM in two Hospitals in France.

**Methods:**

We performed a bicenter retrospective and observational study. All patients treated with AS for SIM between 2014 and 2018 and 2016–2020 were included. The effectiveness of AS was evaluated by parasite clearance, number of deaths, and the length of hospital stay. The real-life safety was assessed by related adverse events (AE) and monitoring of biological blood parameters during the hospital stay and follow-up period.

**Results:**

110 patients were included during the six-year study period. 71.8% of patients were parasite-negative of their day 3 thick and thin blood smears after AS treatment. No patients discontinued AS due to an AE and no serious AE were declared. Two cases of delayed post-artesunate hemolysis occurred and required blood transfusions.

**Conclusion:**

This study highlights effectiveness and safety of AS in non-endemic areas. Administrative procedures must be accelerated in order to obtain full registration and facilitate access to AS in France.

## Introduction

Malaria is one of the deadliest tropical diseases and therefore remains a major public health issue. In 2021, 247 million cases were reported worldwide by the World Health Organization (WHO), and 619 000 patients died of malaria [1]. In non-endemic areas, imported *Plasmodium falciparum* malaria cases continue to occur, so that in 2019 there were 5 540 imported malaria cases reported in France. Among these, severe forms represented 14.8% of imported cases [2]. Since 2010, the WHO established intravenous artesunate (AS) as the first-line treatment in all patients with severe malaria worldwide [3]. Two trials (AQUAMAT, SEQUAMAT) conducted in Africa and Asia respectively, have shown the benefits of AS compared to intravenous quinine in severe malaria. These trials established AS as a first-line treatment for adults and children in endemic countries, and AS was shown to result in a 35% reduction in death rates in adults and a 22.5% reduction in children in Africa [4, 5], as well as a better antiparasitic efficacy due to its ability to act on early circulating ring-stages [6]. A Cochrane review of eight trials enrolling 7 429 patients concluded that there was a total benefit of 39% in terms of mortality reduction in favor of AS (RR 0.61, 95% CI: 0.50 to 0.75) [7]. In addition to the effectiveness profile, AS presents a better safety profile compared to quinine, for which the adverse events have been well described, such as cinchonism, hypoglycemia, and ECG changes [7]. AS was integrated into the therapeutic strategy for severe malaria in 2006 in endemic areas and its use has gradually become widespread, including for travelers returning from these areas. The first AS use in Europe was described in Norway. No deaths, sequelae, or adverse events occurred during the hospital stay among the nine adult patients treated by AS [8]. In 2011, a case series involving 25 patients supported by seven European centers described six cases of post artesunate hemolysis occurring 2–3 weeks after AS treatment. The hemolysis resolved spontaneously in 3 to 6 weeks, although some blood transfusions were required [9]. An 8-year study of severe malaria cases in Europe conducted between 2006 and 2014 detected a 27% incidence of post-artesunate delayed hemolysis (PADH), with a median duration of 14 days (IQR (8–18), and 15% of the patients with PADH received blood transfusions [10]. No PADH cases were reported in either the AQUAMAT or the SEQUAMAT trial. Nevertheless, in these studies the modalities and follow-up data of the patients were not described. In the AQUAMAT trial, only patients with neurological symptoms were followed for 3 to 8 weeks after inclusion.

In non-endemic areas such as Europe, parenteral AS was granted orphan drug status by the European Medicines Agency (EMA) in 2007 [11]. In France, AS has not received marketing authorization and is only available on a compassionate basis, with access granted by the French National Agency for the Safety of Medicines and Health Products (ANSM) since May 2011 [12]. This status allows the national health authorities to control and monitor the use of AS in France as well as a prospective follow-up of imported severe malaria cases by the National Malaria Reference Center.

Since 2011, AS is still being used on a compassionate basis in France despite robust evidence regarding its efficacy and safety in the treatment of severe imported malaria in endemic areas. In non-endemic countries, while P. falciparum is by far the most common causative agent of imported malaria in France and Europe (> 98.5%), AS suffers from slow development due to a lack of full WHO Good Manufacturing Practice (GMP) qualifications as well as the lack of prospective randomized trials in travelers, which are no longer feasible presently for ethical reasons. In the United States, the Food and Drug Administration (FDA) approved AS in May 2020, which allowed artesunate to be made commercially available in hospitals, now free of an expanded-use investigational new drug protocol [13].

After more than 10 years of use in France, AS has not yet received marketing authorization, unlike in Asia and Africa, despite WHO guidelines recommending AS as a first-line treatment of severe imported malaria and several case reports and studies conducted in non-endemic countries. As a result, the only intravenous antimalarial available to treat severe malaria in France is investigational IV artesunate solely available through an expanded-use investigational new drug.

The aim of this retrospective study was to assess the real-life effectiveness and safety of AS in the treatment of severe imported malaria in two University Hospitals in France.

## Patients and methods

### Study population

We performed a bicenter retrospective, observational study that combined data from two French University Hospitals (The Timone University Hospitalin Marseille and the Hôtel Dieu University Hospital in Nantes, France). All patients (pediatric and adult) with severe imported malaria treated by parenteral AS were included. Severe malaria was defined according to the 2014 WHO criteria and national guidelines describing severity criteria [3]. Data were collected from patients treated between January 2014 and June 2018 in the first center (Timone) and between January 2016 to January 2020 for the second center (Hôtel Dieu).

### Outcomes

The primary outcome was assessment of the effectiveness of AS, evaluated by parasite clearance, the number of deaths, and the length of hospital stay. The secondary outcome documentation of the real-life safety, assessed by related adverse events and monitoring of hemoglobin level, transaminase level, blood platelet count, potassium level, and serum creatinine level during the hospital stay and follow-up period.

### Data collection

The data collection method was standardized between the two participating hospitals and compiled in a common database. Data for individual patients were retrospectively extracted from the electronic medical records and the pharmacy computerized system (PHARMA^®^, Computer Engineering *v.5.8* and POWERCHART^®^, Cerner *v.10.0*). All of the patient data were anonymized before analysis. The data collected comprised demographic characteristics, travel history, severity criteria, clinical outcome, length of hospital stay, biological parameters, plasmodium species, parasitemia progression before and under treatment, switch to oral therapy and follow-up. The severity criteria used are those described in the protocol of therapeutic use elaborated by the ANSM and the National Reference Center of Malaria [14]. In this protocol, hyperparasitemia is defined for a rate > 4%, which differs from the WHO definition of 5%. For the study, we used the threshold of hyperparasitemia as defined in the national protocol for access to intravenous artesunate, i.e. 4%. The data were collected during the patients hospital stays and follow-up periods. Due to the retrospective nature of the data collection, some data were missing, mainly regarding follow-up after discharge. Variables were excluded when more than 15% were missing.

### Statistical analysis

Statistical analysis was performed using GraphPad Prism *v8* software. The qualitative and quantitative variables are expressed as numbers (%), means (standard derivation, SD), or medians (interquartile range). We performed descriptive statistics, and comparative analyses were assessed by Fisher’s exact or the Mann-Whitney U test, and we assessed Pearson’s correlation coefficients between data. *P* < 0.05 was considered statistically significant.

## Results

A total of 110 patients received intravenous AS for severe imported malaria during the 6-year study period. Thirty-two patients were identified between January 2014 and June 2018 in the first center and 78 patients between January 2016 and January 2020 in the second center. The overall case-fatality rate was 0.9% (one death) and the median length of stay in the hospital was 4 days [IQR: 3–6]. The demographic data are reported in Table 1. The patients from the two centers did not differ in terms of the mean [SD] age (37.0 [19.7] vs. 42.1 [20.3]; *p = 0.26*) and sex (34% vs. 29% females; *p = 0.43*). Almost two-thirds of the patients were male (69.1%). All of the patients acquired their malaria infection on the African continent, except for one patient who contracted malaria in Amazonia. The majority of cases resulted from return from Western Africa (64.5%) and Central Africa (31%), with three main countries: Ivory Coast (n = 30), Cameroon (n = 22), and Guinea (n = 19). Twenty-two patients (20%) followed a malaria prophylaxis treatment but only 6 (5.5%) were compliant with their treatment.

The average time between the onset of symptoms and the return was 9 days [median, IQR: 4.5–15]. For 80% of the patients in this series, this was their first malaria episode, while for 21 patients (19.1%), a previous history of having had malaria could be documented. Despite AS having received institutional authorization exclusively for treatment of *Plasmodium falciparum* species malaria, two patients were treated for *P. ovale* malaria and one patient for *P. vivax* malaria, following a first reading identifying a *P. falciparum*. Each patient was then treated with the appropriate oral molecule following the identification of the species on second reading.

**Table 1:** Demographic data of the patients with severe imported malaria

Parameters	All (n = 110)
Gender	
Male (%) Female (%)	76 (69.1) 34 (30.9)
Age (years)	
Median age (IQR) Age ≤ 18 years (%) Age ≥ 60 years (%)	38.5 (23.8–57) 22 (20) 21 (19.1)
Weight (kg)	
Median weight (IQR)	71 (59–90)
Malaria acquisition region	
Western africa (%) Central Africa (%) East Africa (%) North Africa (%) Non-african (%)	71 (64.5) 34 (31.0) 3 (2.7) 1 (0.9) 1 (0.9)
Chemoprophylaxis	
No (%) Yes but incomplete (%) Yes (%) Unknown (%)	86 (78.2) 16 (14.5) 6 (5.5) 2 (1.8)
Time from return and onset of symptoms (days)	
Median time (IQR) ≤ 3 (%) 4–7 (%) > 7 (%) Missing data (%)	9 (4.5–15) 24 (21.8) 26 (23.6) 59 (53.6) 1 (1.0)
Plasmodium type	
*Falciparum (%)* *Ovale (%)* *Vivax (%)*	107 (97.3) 2 (1.8) 1 (0.9)
Malaria infection history	
Primary infection (%) Reinfection (%) Missing data (%)	88 (80) 21 (19.1) 1 (0.9)

Almost three-quarters of the patients (71.8%) were parasite-negative of their day 3 blood smears after AS treatment. Data were missing for 13 patients. Of the 17 patients with a positive thick and thin blood smears at D3 (15.5%), 12 were parasite-negative on D7, two on D10, and3 remained without follow-up data. There were no documented early or late parasitological failures. One patient with four severity criteria at admission died within the first three days after hospital management. However, this patient suffered from severe hepatic impairment with a high risk of severe bleeding due to an active pancreatic carcinoma.

The clinical parameters, severe malaria characteristics and treatment modalities are detailed in Table 2. The median baseline hemoglobin (Hb) level at admission was 12.5 g/dL [IQR: 10.8–14.3] and the median baseline parasitemia was 5.6% [IQR: 1.5–10]. The median number of severity criteria was 2 (1–3). Hyperparasitemia (> 4%) was the most common severity criterion, involving more than half of the patients (53.6%), followed by jaundice (39.1%) and hyperlactatemia (25.5%). Twenty-three patients (20.9%) only had isolated hyperparasitemia. The majority of severe malaria cases were defined with a single severity criterion (41%) or two criteria (22.7%), justifying AS introduction, while 5.4% (6/110) met fiveor more criteria. Intravenous artesunate was used as first-line treatment in the majority of cases (79.1%) but also in second-line for 22 patients due to deterioration of the clinical condition under treatment with quinine (n = 4) or oral therapy (n = 18) and progression to severe malaria with severity criteria. The first-line oral therapies were artemether/lumefantrine 20 mg/120 mg (Novartis Pharma SAS), atovaquone/proguanil 250 mg/100 mg (GlaxoSmithKline), and mefloquine 250 mg (Cheplapharm). The median number of AS doses was three [IQR: 3–4] and all adults patients received a dose of 2.4 mg/kg, while thepediatric patients aged1 to 5 years and who weighed less than 20 kg (n = 5) received a dose of 3 mg/kg. The follow-on treatments by oral drug after intravenous therapy are summarized in Table 2. Piperaquine/artenimol 320 mg/4 0 mg (Alfasigma SpA) (42.7%) was the treatment of choice for oral relay, followed by artemether/lumefantrine 20 mg/120 mg (Novartis Pharma SAS) (29.1%) and atovaquone/proguanil 250 mg/100 mg (GlaxoSmithKline) (21%).

**Table 2:** Clinical parameters, malaria severity criteria, and therapeutic management of patients

Parameters	All (n = 110)
**Length of hospital stay** [median, IQR] **Overall case fatality rate** (%)	4 [3–6] 1 (0.9)
**Clinical characteristics**	
Median hemoglobin at admission [IQR] Median parasitemia, % [IQR]	12.5 [10.8–14.3] 5.6 [1.5–10]
**Severe malaria description**	
Number of severe malaria criteria [median, IQR] 1 (%) 2 (%) 3 (%) 4 (%) 5 (%) > 5 (%) Missing data (%) Identified severity criteria (%) Hyperparasitemia (> 4%) Jaundice Hyperlactatemia Impaired consciousness/coma Acute renal failure (creat > 265 µmol/L) Macroscopic hemoglobinuria Respiratory failure Circulatory collapse/shock Acidosis Anaemia < 7 g/dL Multiple convulsions Bleeding Hypoglycemia (< 2.2 mmol/L)	2 [1–3] 45 (41) 25 (22,7) 20 (18.2) 11 (10) 3 (2.7) 3 (2.7) 3 (2.7) 59 (53.6) 43 (39.1) 28 (25.5) 24 (21.8) 18 (16.4) 16 (14.5) 12 (10.9) 12 (10.9) 10 (9.1) 7 (6.4) 2 (1.8) 2 (1.8) 0 (0)
**Treatment management** Artesunate use	
First line (%) Second line (%) Missing data (%) Median number of AS doses [IQR]	87 (79.1) 22 (20) 1 (0.9) 3 [3.4]
Switch to oral therapy	
Piperaquine/artenimol (%) Artemether/lumefantrine (%) Atovaquone/proguanil (%) Chloroquine (%) None (%) Unknown (%)	47 (42.7) 32 (29.1) 23 (21) 1 (0.9) 2 (1.8) 5 (4.5)
Negative parasitemia 72 h after AS initiation	
Yes (%) No (%) Missing data (%) Death (%)	79 (71.8) 17 (15.5) 13 (11.8) 1 (0.9)

Patient follow-up information was available for only one of the two centers. Of these 78 patients, 61 (78.2%) had follow-up consultations as part of their post-treatment monitoring with AS. These consultations were mostly scheduled between D7 and D28 and extended to D60 for two cases. For 44.3% of the patients there weretwo consultationson approximately D7 and D28, while for 23 patients (37.7%) there was only one consultation. Five patients did not attend the scheduled consultations and three were followed up in the context of their return abroad after hospitalization. Two hospitalized patients in wards with few malaria patients (nephrology and pediatric units) did not have a scheduled follow-up consultation.

The adverse drug reactions are shown in Table 3.

**Table 3:** Adverse drug reactions

Parameters	All (n = 110)
Mean decrease Hb level at day 3, g/dL [SD]	-2.17 [1.42]
**Adverse drug events (%)**	
Anemia, Hb < 10.5 g/dL (%) WHO grade 1 (9.5 < Hb < 10.5) WHO grade 2 (8 < Hb < 9.5) WHO grade 3 (6.5 < Hb < 8) Hepatic disorders (transaminases elevation) (%) Abnormal kalemia, < 3.4 or > 4.5 mmol/L (%) Creatinine level > 265 µmol/L (%) Abnormal platelet count, < 150 or > 400 G/L (%)	44 (40.4) 19 (43.2) 17 (38.6) 8 (18.2) 21 (19.3) 14 (12.8) 2 (1.8) 0 (0)

None of the patients discontinued AS due to an adverse event, and no serious adverse events, or cutaneous or injection site reaction were declared. Sixty-three patients (57.3%) presented a biological disorder during the 7-day follow-up of AS therapy, without a major clinical impact or requirement for readmission, except for two patients. The main biological adverse event was post-AS anemia. On day 7 after hospital admission, 44 patients had anemia according to the WHO guidelines, with mild anemia for 43.2% of them (WHO grade 1). Three patients suffered from severe anemia (WHO grade 3). Of these, half (24/44) already had anemia on hospital admission, with a mean decrease in the Hb level of – 0.94 g/dL (SD: 2.8). On day 3 after admission and AS therapy, the Hb level decreased by an average of – 2.17 g/dL (SD: 1.42). Two cases of delayed hemolysis were detected during outpatient follow-up. The first patient had a Hb level of 6.1 g/dL and lactate dehydrogenase (LDH) at five times normal on day 17 after treatment initiation, without a clinical impact except for mucocutaneous pallor (admission level: 13.3 g/dL and 10.1 g/dL at hospital discharge). The parasitemia level was3%, and four AS doses were administered followed by an oral relay with artemether/lumefantrine (Novartis Pharma SAS). The patient’s care for delayed hemolysis was readmission as an outpatientfor blood transfusion (two units of packed red blood cells). PADH was reported in the national pharmacovigilance database. The second patient presented with hyperparasitemia at 18% and exhibited four severity criteria: acidosis, hyperlactatemia, acute renal failure and jaundice. The baseline creatinine level at admission was 477 µmol/L. Six AS doses were administered with oral relay on day 5 with piperaquine/artenimol (Alfasigma SpA) (3 days). During the hospital stay, their Hb levels decreased from 8.9 g/dL at baseline to 6.7 g/dL on day 3 and reached a nadir of 5.3 on day 12. After blood transfusion, their Hb level stabilized at 7.4 g/dL and became 8.6 g/dL at discharge on day 23. At a follow-up visit at day 60, their Hb level was 12 g/dL, with slightly elevated LDH. The parasitemia was negative on day 3 and day 7. At the same time, the patient suffered from multi-organ failure within three days of admission, with an increase in creatinine to 611 µmol/L associated with encephalopathy (hyperuricemia). The patient started hemodialysis on day 5 for two days. At discharge (D18), their creatinine level was 147 µmol/L and decreased to 83 µmol/L at the 28-day follow-up consultation. This patient was the only one who exhibited elevated creatinine levels at discharge among the 18 patients with acute renal failure at admission. In total, two patients had elevated creatinine levels after AS therapy. One patient reported neuropathy of both feet that spontaneously resolved and could be attributed to oral antimalarial therapy. An abnormal potassium level was detected for 14 patients (12.8%) during AS treatment, mainly hyperkaliemia (mean: 4.9 mmol/L, SD = 0.23).

**Fig. 1:**
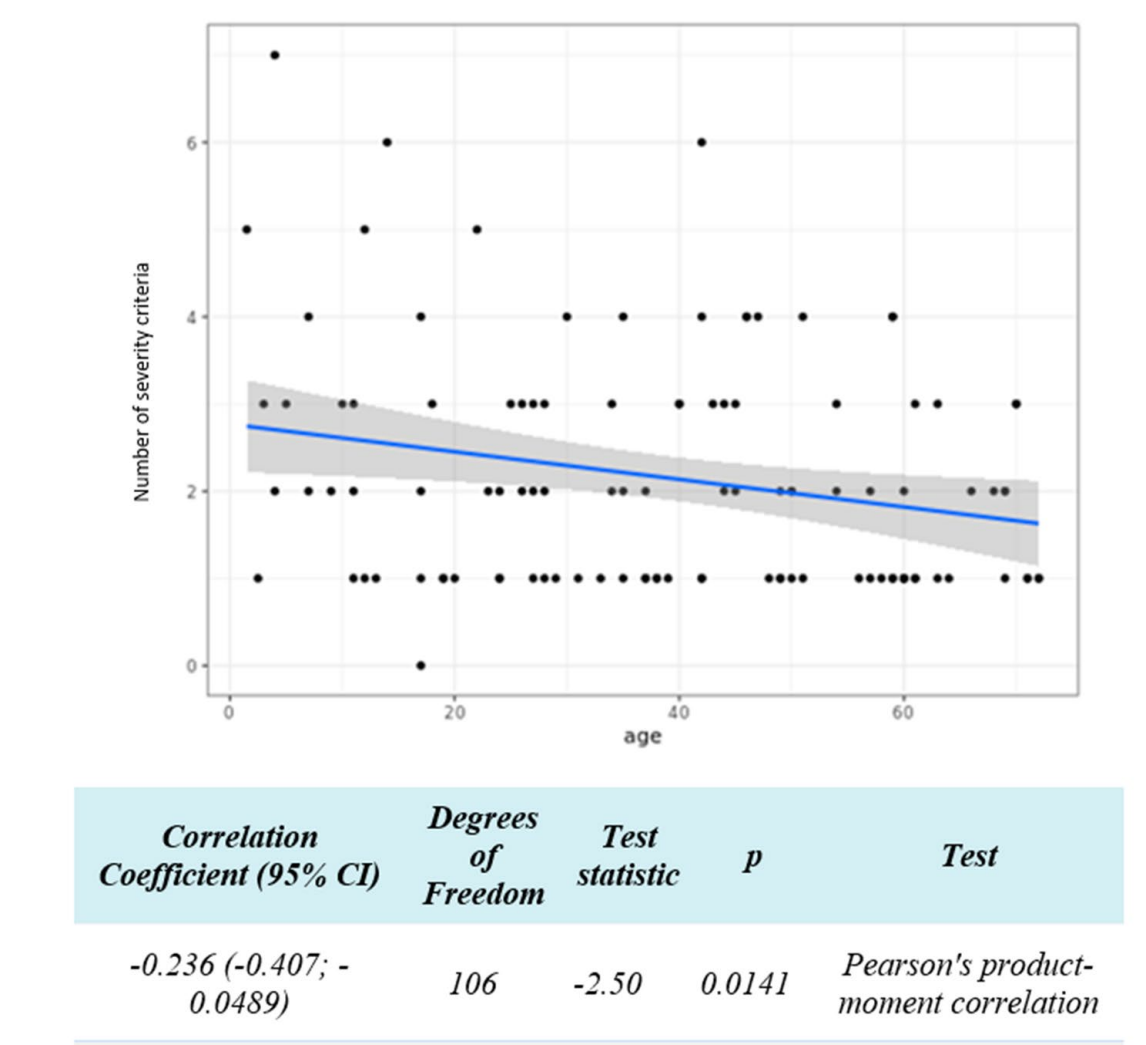
Univariate analysis of the number of severity criteria according to age

**Fig. 2:**
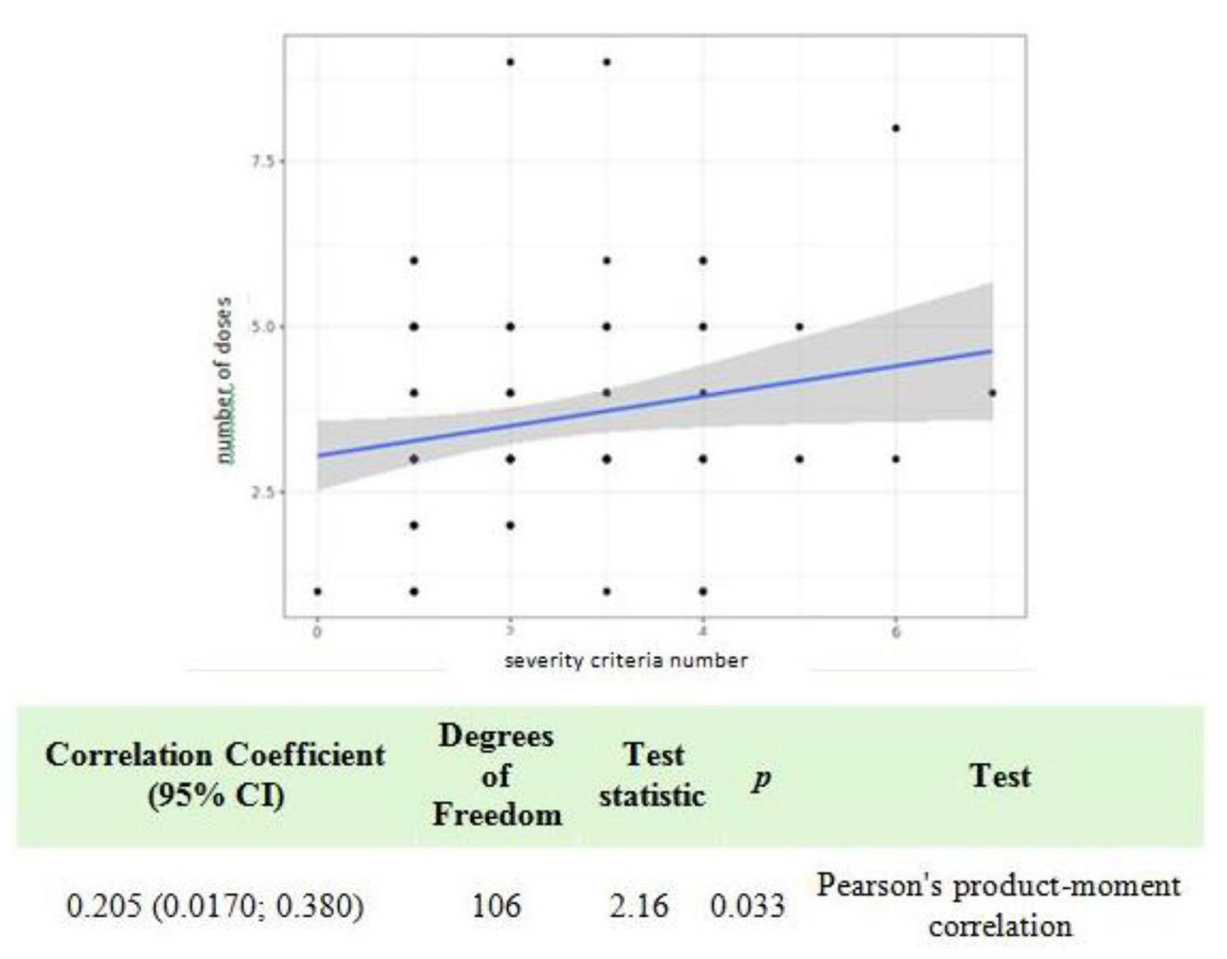
*Univariate analysis of the number of AS doses according to the number of severity criteria*

**Fig. 3:**
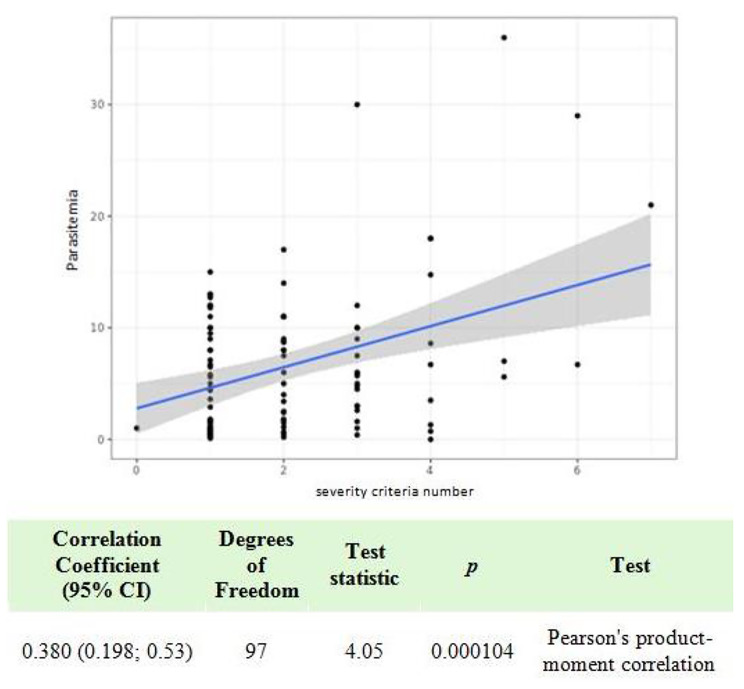
Univariate analysis of the parasitemia distribution according to the number of severity criteria

Table 4 identifies the risk factors associated with the number of AS doses and the consequences of reinfection. The analysis showed a linear correlation between age and the number of severity criteria (*p* < 0.014, Fig. 1). There was no difference between patients exhibiting malaria reinfection versus primary infection in terms of the number of severity criteria (*p* = 0.52) and no difference in terms of the parasitemia level (*p* = 0.71). An increase in the number of severity criteria corresponded with an increase in the number of AS doses administrered (significant correlation *p* = 0.033, Fig. 2). The parasitemia level also significantly correlated with the number of severity criteria (*p* < 0.001, Fig. 3). There was an association between the number of AS doses and abnormal creatinine levels (*p* < 0.015), but there was not a relationship with anemia secondary effects (*p* = 0.15) or hepatic transaminases levels (*p* = 0.28).

**Table 4:** Identified risk factors for the number of AS doses and the consequences of reinfection

Associated risk factors	Yes	No	*P* value
**AS dose number (mean, SD)**			
Anemia, Hb < 10.5 g/dL Hepatic disorders (transaminases elevation) Creatinine level > 265 µmol/L	3.77 (1.60) 3.71 (1.15) 7.00 (1.41)	3.34 (1.37) 3.48 (1.55) 3.46 (1.41)	0.15 0.28 0.015
**Reinfection (mean, SD)**			
Parasitemia Severity criteria number	6.43 (6.93) 2.00 (1.26)	6.82 (6.53) 2.20 (1.37)	0.71 0.52

## Discussion

This retrospective, observational bicenter study presents additional data for six-years of real-life use of AS in two University Hospital centers in France. This work reports the solid effectiveness and reassuring safety data of intravenous AS in the treatment of severe malaria in non-endemic areas. No recrudescence was reported, and only two cases of PADH were recorded among the 110 patients of both participating centers.

The efficacy of intravenous artesunate is well established in endemic areas and has shown superiority in antiparasitic efficacy and survival compared to quinine, thereby making AS a first-line treatment for severe malaria [3–5]. Despite the lack of controlled and randomized trials investigating the superiority of AS versus quinine in non-endemic areas for ethical reasons, several studies have confirmed the efficacy and good tolerability of AS in countries with a high level of care [3]. The mortality rate is very low and ranges between 4% and 15% in the studies to date [10, 15–19]. A systematic review of AS use in non-endemic areas found a mortality rate of 4% [20] and is in keeping with the prospective study conducted by Jaureguyberry et al., which reported a mortality rate of 5% [21]. In our study, only one patient died, and this was a patient who also had active cancer.

The main arguments in favor of superior efficiency of AS are the benefit derived from reducing the parasite clearance time, particularly in hyperparasitemic patients (> 10%), as well as the reduction in the length of stay in intensive care units (ICU) and hospital treatments in European patients [10, 22].

Reduction of the parasitic clearance time has been described by Kurth et al. and *Eder et al.* with respectively 21 h and 20 h in favor of AS [22, 23].This relates to the broader parasitical action of AS compounds. Unlike quinine, artesunate acts on almost all intraerythrocytic parasites and in particular on young circulating forms explaining the rapid parasiticidiosis. AS also prevents sequestration and amplifies natural spleen action (pitting). While the use of AS does not reduce hospital the length of stay, it does reduce the ICU stay (HR = 1.18, CI 95% [1.02–1.36], leading to cost-effective use of AS compared to quinine [24]. The cost-effectiveness of AS was also demonstrated in an economic evaluation conducted in South Asia [21].

In our study, no parasitic failure or recrudescence were documented. In its 5-year synthesis report of AS use, the French national reference center reported a mortality rate of 3% (28/919) and nine cases (1%) of parasitic failure including two recrudescence cases (at day 24 and day 28 of treatment). Failure was defined as persistent circulating trophozoites at day 7 [25].

Only two patients had a documented PADH in this study, and no serious adverse reactions were reported. These results support the good tolerance of AS in real life and are in line with the data described in other studies. However, not all patients in our cohort were systematically followed up during the 4-week post-treatment period. It is, therefore, possible that these results suffer from a degree of bias due to under-reporting. Rolling et al. demonstrated a better tolerance of AS compared to quinine, with delayed hemolysis observed for 60% of patients as well as temporary deterioration of renal function (60%) while quinine caused at least one adverse reaction in 71% of patients. Quinine is known to havea number of adverse effects such as hypoglycemia, hearing disturbances, cardiotoxicity, and cinchonism [26]. PADH is the most frequently reported adverse event associated with AS use and is not described with quinine. PADH, by definition, associates a hemoglobin decrease > 10% combined with a rise in the median LDH > 10% at day 8 after AS treatment initiation [27]. The first case of PADH was described in Japan in 2002 [28]. In the literature, PADH rates range between 20% and 30% and occurr within 2 to 6 weeks after AS initiation [9, 21, 29]. A review conducted in 2015 identified a delayed hemolysis in 7–21% of patients treated by AS [30], while a systematic review found an occurrence rate of 15.3%, of which 50% required a blood transfusion. The hemolysis corrected spontaneously within a few days to a few weeks, and no sequelae were identified after the hemolysis episode [20]. Hyperparasitemia has been identified as a risk factor for delayed hemolysis in travelers as well as people of young age in endemic countries [26, 30]. The origin of hemolysis remains poorly defined, but several mechanisms have been proposed. The pitting process exerted by artemisinin compounds is one of the reasons put forward. Patients with a high proportion of pitted red blood cells have an increased risk of PADH as a result of their late elimination [31, 32]. However, it is important to differentiate between hemolysis caused by biliary hemoglobinuria fever (hemolysis with malaria-associated hemoglobinuria) as well as severe hemolysis caused by malaria itself and the PADH process. On the other hand, anemia caused by severe malaria may, in some patients, slowly return to basal levels, without delayed hemolysis activity. This PADH risk calls for follow-up of patients for a period of four weeks after treatment with AS, as recommended by the WHO [3]. Acute renal failure is another adverse event observed with artesunate, although, kidney failure may be due to the malaria itself. In our study, there was an association between the number of AS doses received and abnormal creatinine levels. This is probably a result of the fact that severe patients, including those with renal failure, require longer treatments. Two case reports showed a diuretic effect of AS without a decrease in renal function [26, 33].

The present study has several limitations. Adverse events might be underreported due to the retrospective review of the medical charts as well as due to data based on patient self-reporting of information. Moreover, some data may be incomplete due to the sometimes incomplete follow-up of discharged patients (follow-up at four weeks). As a result, not all patients were systematically screened and followed during the four weeks post-treatment with artesunate, implying a likely degree of underreporting of PADH. In our study, comorbidities and length of stay were not collected. Also, LDH levels were not collected for the PADH description. Finally, the retrospective nature of the study does not guarantee the completeness of the data collected despite the consultation of electronic medical records and some data were missing, mainly regarding follow-up after discharge.

In high level of care areas, AS has gradually replaced quinine since 2011. Nevertheless differences in access to AS in Europe have led to disparate recommendations. Several studies have demonstrated the efficacy and safety of AS for severe malaria treatment with a faster ICU discharge rate and cost-effective therapy in high level of care areas [24]. In the United States, the FDA approved AS in May 2020, thus ending the expanded-use investigational new drug protocol and allowing AS to be commercially available in hospitals. [13]. In France, a monitoring group has been set up and issues regular reports on the use of AS under the aegis of ANSM. The last report involving five years of use described a cohort of 919 patients with 3% mortality and 1% parasitological failure. One hundred and nine patients (11.8%) experienced an adverse event, which for 59% represented an undesirable anemic effect and 25 PADH were reported (2.7%). Currently, AS is the only intravenous antimalarial available to treat severe malaria in France solely available through a compassionate use authorization program in France since 2011, involving administrative procedures, agreement of the ANSM for the initiation of the treatment, tedious prescribing, and time-consuming management of the treatment by the hospital pharmacy. This monitoring system is showing its limitations given the increasing number of patients being treated and in light of the urgency of the clinical situation and the abundance of reassuring data regarding the safety of AS. All these elements contrast with the recommendations for the management of severe malaria, which place AS in first-line in non-endemic areas, but also with the reassuring safety profile of the product. Finally, since 2011, the National Reference Center for Malaria and the ANSM have been collecting data from patients who have received artesunate, which now provides sufficient hindsight regarding use of this treatment.

## Conclusion

In conclusion, this work highlights the effectiveness and safety of intravenous AS in non-endemic areas. In non-endemic countries, AS suffers from slow development due to the lack of full WHO Good Manufacturing Practice (GMP) qualification as well as the lack of prospective randomized trials in travelers which are no longer possible for ethical reasons.

It is, therefore, essential that the administrative procedures that have been underway for more than 10 years are accelerated in order to obtain full registration, which will facilitate access to AS in France.

The current administrative burden for the initiation of treatment is not in line with the use of AS as an emergency and first-line treatment for severe imported malaria and as a cost-effective therapeutic strategy in high-care areas. The data regularly collected by the national reference center and the ANSM since 2011 should help accelerate this process. Finally, the recent overhaul in 2021 of the French system of treatments under compassionate access aimed at simplifying access to these treatments, particularly for manufacturers, is an opportunity to accelerate the procedures underway in order to obtain full approval and widespread access to intravenous artesunate in France.

## Data Availability

The datasets used and/or analysed during the current study are available from the corresponding author on reasonable request.
